# Dichlorido-1κ*Cl*,3κ*Cl*-hexa­kis­[1,1,2,2,3,3(η^5^)-cyclo­penta­dien­yl]di-μ_2_-oxido-1:2κ^2^ *O*:*O*;2:3κ^2^ *O*:*O*-trizirconium(IV)

**DOI:** 10.1107/S1600536812024968

**Published:** 2012-06-13

**Authors:** Bradley M. Kraft, William W. Brennessel

**Affiliations:** a Department of Chemistry, St. John Fisher College, Rochester, NY 14618, USA; b Department of Chemistry, University of Rochester, Rochester, NY 14627, USA

## Abstract

The title compound, [Zr_3_(C_5_H_5_)_6_Cl_2_O_2_], exists as discrete mol­ecules possessing a series of three Cp_2_Zr units (Cp is cyclo­penta­dien­yl) bridged by oxide ligands and end-capped by chloride ligands. The Cp planes in the central and terminal zirconocene units form dihedral angles of 53.3 (2) and 53.5 (2)°, respectively. The two Zr—O—Zr bridge angles are nearly linear and form a planar Zr_3_O_2_ core. The mol­ecule bears *C*2 symmetry with the central Zr atom lying on a crystallographic twofold axis.

## Related literature

For closely related Zr mol­ecules with only one oxo bridge, see: Reid *et al.* (1965 ▶); Clarke & Drew (1974 ▶); Kuz’mina *et al.* (1988 ▶); Nieger *et al.* (1999 ▶); Spletstoser *et al.* (2007 ▶). For cyclic trimeric oxozirconocenes, see: Arnold *et al.* (2011 ▶); Boutonnet *et al.* (1995 ▶); Mikhailova *et al.* (1993 ▶). For similar structures with terminal Zr–Cl bonds, see: Corey *et al.* (1995 ▶); Reddy & Petersen (1989 ▶). For the Hf analog, but with methyl-substituted cyclo­penta­dienyl rings, see: Wisniewska *et al.* (2008 ▶).
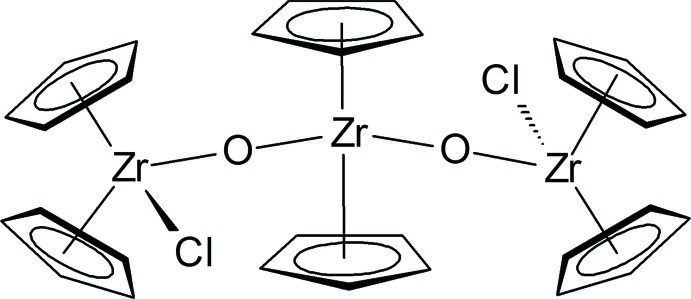


## Experimental

### 

#### Crystal data

[Zr_3_(C_5_H_5_)_6_Cl_2_O_2_]
*M* *_r_* = 767.10
Orthorhombic, 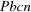

*a* = 7.8809 (4) Å
*b* = 18.0518 (10) Å
*c* = 20.1883 (11) Å
*V* = 2872.1 (3) Å^3^
*Z* = 4
Mo *K*α radiation
μ = 1.28 mm^−1^
*T* = 223 K
0.20 × 0.18 × 0.04 mm

#### Data collection

Bruker SMART APEXII CCD Platform diffractometer
Absorption correction: multi-scan (*SADABS*; Sheldrick, 2008*b* ▶) *T*_min_ = 0.784, *T*_max_ = 0.951
34715 measured reflections
4032 independent reflections
2569 reflections with *I* > 2σ(*I*)
*R*_int_ = 0.099

#### Refinement

*R*[*F* ^2^ > 2σ(*F* ^2^)] = 0.040
*wR*(*F* ^2^) = 0.100
*S* = 1.01
4032 reflections
168 parameters
H-atom parameters constrained
Δρ_max_ = 0.52 e Å^−3^
Δρ_min_ = −0.48 e Å^−3^

### 

Data collection: *APEX2* (Bruker, 2011 ▶); cell refinement: *SAINT* (Bruker, 2009 ▶); data reduction: *SAINT*; program(s) used to solve structure: *SIR97* (Altomare *et al.*, 1999 ▶); program(s) used to refine structure: *SHELXL97* (Sheldrick, 2008*a* ▶); molecular graphics: *SHELXTL* (Sheldrick, 2008*a* ▶); software used to prepare material for publication: *SHELXTL*.

## Supplementary Material

Crystal structure: contains datablock(s) I, global. DOI: 10.1107/S1600536812024968/pk2418sup1.cif

Structure factors: contains datablock(s) I. DOI: 10.1107/S1600536812024968/pk2418Isup2.hkl

Additional supplementary materials:  crystallographic information; 3D view; checkCIF report

## Figures and Tables

**Table:** 

Zr1—O1	1.921 (3)
Zr1—Cl1	2.4857 (12)
Zr2—O1	1.980 (3)
Zr2—O1^i^	1.980 (2)

**Table:** 

O1—Zr1—Cl1	97.23 (8)
O1—Zr2—O1^i^	102.43 (15)
Zr1—O1—Zr2	171.43 (15)

Symmetry code: (i) 
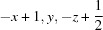
.

**Fig. 1.**
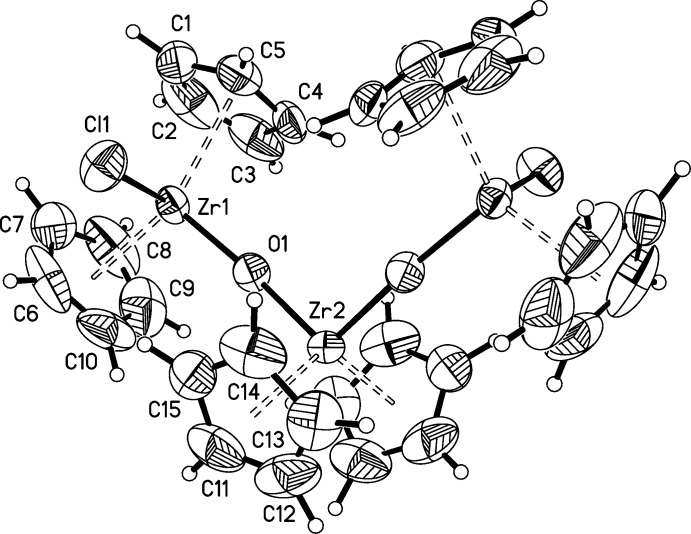
A displacement ellipsoid (50% probability) drawing. Symmetry equivalent atoms generated by a crystallographic twofold axis that includes atom Zr2.

## supplementary crystallographic information

### Comment

The geometry around each Zr in the title compound is pseudotetrahedral with the center of each Cp ligand taken as a single coordination site. The dihedral angle between the Cp rings in the central zirconocene unit is 53.3 (2)° and that between the Cp rings in the terminal zirconocene units is 53.5 (2)°, similar to those in related structures (Mikhailova *et al.*, 1993, Spletstoser *et al.*, 2007). As with many cyclic trimeric oxozirconocenes (Arnold *et al.*, 2011; Boutonnet *et al.*, 1995; Mikhailova *et al.*, 1993), the three Zr atoms and bridging O atoms are also planar in this open structure with the µ_2_-O ligands deviating above and below the plane each by 0.144 (3) Å. The nearly linear Zr–O–Zr angles (171.43 (15)°) indicate double-bonding character with each neighboring Zr atom. The O–Zr–O angle is 102.43 (15)°, which is wider than that found in cyclic trinuclear oxozirconocenes (Arnold *et al.*, 2011; Mikhailova *et al.*, 1993) and wider than that in the methyl-substituted cyclopentadienyl hafnium analog (Wisniewska *et al.*, 2008). The Zr–O distances of 1.921 (3) and 1.980 (2) Å are comparable with those of other µ_2_-oxo Zr complexes (Kuz'mina *et al.*, 1988, Spletstoser *et al.*, 2007). The Zr–Cl distances of 2.4857 (12) Å are typical (Corey *et al.*, 1995; Reddy & Petersen, 1989).

### Experimental

The title compound was isolated as pale yellow needles upon hydrolysis of Cp_2_Zr(Cl)(*L*) [*L* = 4-methyl-2,6-bis(2,6-diisopropylphenylimino)phenoxy] by adventitious water in THF/pentane.

### Crystal data

**Table:**

[Zr_3_(C_5_H_5_)_6_Cl_2_O_2_]	*F*(000) = 1520
*M**_r_* = 767.10	*D*_x_ = 1.774 Mg m^−^^3^
Orthorhombic, *P**b**c**n*	Mo *K*α radiation, λ = 0.71073 Å
Hall symbol: -P 2n 2ab	Cell parameters from 4069 reflections
*a* = 7.8809 (4) Å	θ = 2.5–27.2°
*b* = 18.0518 (10) Å	µ = 1.28 mm^−^^1^
*c* = 20.1883 (11) Å	*T* = 223 K
*V* = 2872.1 (3) Å^3^	Needle, pale yellow
*Z* = 4	0.20 × 0.18 × 0.04 mm

### Data collection

**Table:**

Bruker SMART APEXII CCD Platform diffractometer	4032 independent reflections
Radiation source: fine-focus sealed tube	2569 reflections with *I* > 2σ(*I*)
Graphite monochromator	*R*_int_ = 0.099
area detector, ω scans per φ	θ_max_ = 29.6°, θ_min_ = 2.0°
Absorption correction: multi-scan (*SADABS*; Sheldrick, 2008*b*)	*h* = −10→10
*T*_min_ = 0.784, *T*_max_ = 0.951	*k* = −25→25
34715 measured reflections	*l* = −27→27

### Refinement

**Table:**

Refinement on *F*^2^	Primary atom site location: structure-invariant direct methods
Least-squares matrix: full	Secondary atom site location: difference Fourier map
*R*[*F*^2^ > 2σ(*F*^2^)] = 0.040	Hydrogen site location: inferred from neighbouring sites
*wR*(*F*^2^) = 0.100	H-atom parameters constrained
*S* = 1.01	*w* = 1/[σ^2^(*F*_o_^2^) + (0.0427*P*)^2^ + 1.0879*P*] where *P* = (*F*_o_^2^ + 2*F*_c_^2^)/3
4032 reflections	(Δ/σ)_max_ = 0.001
168 parameters	Δρ_max_ = 0.52 e Å^−^^3^
0 restraints	Δρ_min_ = −0.48 e Å^−^^3^

### Special details

**Table:**

Geometry. All e.s.d.'s (except the e.s.d. in the dihedral angle between two l.s. planes) are estimated using the full covariance matrix. The cell e.s.d.'s are taken into account individually in the estimation of e.s.d.'s in distances, angles and torsion angles; correlations between e.s.d.'s in cell parameters are only used when they are defined by crystal symmetry. An approximate (isotropic) treatment of cell e.s.d.'s is used for estimating e.s.d.'s involving l.s. planes.
Refinement. Refinement of *F*^2^ against ALL reflections. The weighted *R*-factor *wR* and goodness of fit *S* are based on *F*^2^, conventional *R*-factors *R* are based on *F*, with *F* set to zero for negative *F*^2^. The threshold expression of *F*^2^ > σ(*F*^2^) is used only for calculating *R*-factors(gt) *etc*. and is not relevant to the choice of reflections for refinement. *R*-factors based on *F*^2^ are statistically about twice as large as those based on *F*, and *R*- factors based on ALL data will be even larger.

### Fractional atomic coordinates and isotropic or equivalent isotropic displacement parameters (Å^2^)

**Table:**

	*x*	*y*	*z*	*U*_iso_*/*U*_eq_	
Zr1	0.65131 (4)	0.40000 (2)	0.110865 (18)	0.03829 (12)	
Zr2	0.5000	0.26636 (3)	0.2500	0.03404 (13)	
Cl1	0.35698 (14)	0.43025 (7)	0.07584 (6)	0.0618 (3)	
O1	0.5931 (3)	0.33507 (14)	0.18272 (12)	0.0423 (6)	
C1	0.6980 (11)	0.5393 (3)	0.1109 (3)	0.086 (2)	
H1	0.6515	0.5671	0.0760	0.103*	
C2	0.8558 (11)	0.5081 (4)	0.1121 (4)	0.104 (3)	
H2	0.9373	0.5115	0.0783	0.125*	
C3	0.8755 (7)	0.4714 (3)	0.1705 (4)	0.0807 (18)	
H3	0.9715	0.4440	0.1833	0.097*	
C4	0.7291 (7)	0.4814 (2)	0.2081 (2)	0.0576 (12)	
H4	0.7095	0.4634	0.2511	0.069*	
C5	0.6183 (6)	0.5226 (2)	0.1703 (2)	0.0596 (12)	
H5	0.5080	0.5368	0.1826	0.071*	
C6	0.6391 (7)	0.3283 (5)	0.0034 (3)	0.092 (2)	
H6	0.5346	0.3266	−0.0191	0.110*	
C7	0.7644 (11)	0.3813 (3)	−0.0040 (3)	0.089 (2)	
H7	0.7613	0.4229	−0.0320	0.107*	
C8	0.8942 (7)	0.3612 (5)	0.0376 (4)	0.094 (2)	
H8	0.9985	0.3858	0.0425	0.113*	
C9	0.8456 (10)	0.3002 (5)	0.0700 (3)	0.096 (2)	
H9	0.9095	0.2760	0.1028	0.115*	
C10	0.6963 (11)	0.2792 (3)	0.0493 (3)	0.089 (2)	
H10	0.6376	0.2369	0.0639	0.107*	
C11	0.3707 (7)	0.1773 (3)	0.1673 (3)	0.0814 (17)	
H11	0.4466	0.1509	0.1402	0.098*	
C12	0.3137 (8)	0.1554 (3)	0.2279 (4)	0.0832 (18)	
H12	0.3419	0.1105	0.2486	0.100*	
C13	0.2116 (7)	0.2073 (3)	0.2536 (3)	0.0727 (14)	
H13	0.1567	0.2054	0.2950	0.087*	
C14	0.2019 (6)	0.2661 (3)	0.2063 (3)	0.0775 (17)	
H14	0.1417	0.3107	0.2106	0.093*	
C15	0.2988 (7)	0.2439 (3)	0.1529 (3)	0.0649 (13)	
H15	0.3129	0.2705	0.1132	0.078*	

### Atomic displacement parameters (Å^2^)

**Table:**

	*U*^11^	*U*^22^	*U*^33^	*U*^12^	*U*^13^	*U*^23^
Zr1	0.0372 (2)	0.0449 (2)	0.03279 (19)	−0.01004 (17)	0.00453 (16)	−0.00460 (16)
Zr2	0.0321 (2)	0.0297 (2)	0.0403 (3)	0.000	−0.0004 (2)	0.000
Cl1	0.0478 (6)	0.0725 (7)	0.0651 (7)	−0.0033 (6)	−0.0076 (6)	0.0129 (6)
O1	0.0402 (15)	0.0460 (14)	0.0407 (15)	−0.0082 (12)	0.0036 (12)	−0.0024 (12)
C1	0.154 (7)	0.049 (3)	0.054 (3)	−0.038 (4)	−0.011 (4)	0.009 (2)
C2	0.119 (6)	0.098 (5)	0.095 (5)	−0.073 (5)	0.052 (5)	−0.041 (4)
C3	0.052 (3)	0.078 (4)	0.112 (5)	−0.009 (3)	−0.018 (3)	−0.044 (4)
C4	0.079 (3)	0.055 (3)	0.039 (2)	−0.019 (2)	−0.009 (2)	−0.011 (2)
C5	0.067 (3)	0.049 (2)	0.062 (3)	−0.005 (2)	0.005 (3)	−0.013 (2)
C6	0.060 (4)	0.143 (6)	0.071 (4)	0.008 (4)	−0.010 (3)	−0.064 (4)
C7	0.146 (7)	0.076 (4)	0.044 (3)	0.009 (4)	0.041 (4)	−0.003 (3)
C8	0.051 (3)	0.136 (6)	0.096 (5)	−0.027 (4)	0.036 (3)	−0.057 (5)
C9	0.091 (5)	0.126 (6)	0.071 (4)	0.047 (5)	0.009 (4)	−0.022 (4)
C10	0.119 (6)	0.058 (3)	0.089 (5)	−0.018 (4)	0.053 (4)	−0.032 (3)
C11	0.079 (4)	0.066 (3)	0.100 (5)	−0.016 (3)	−0.020 (3)	−0.029 (3)
C12	0.069 (4)	0.057 (3)	0.124 (6)	−0.026 (3)	−0.021 (4)	0.017 (3)
C13	0.045 (3)	0.094 (4)	0.079 (4)	−0.024 (3)	0.002 (3)	0.006 (3)
C14	0.039 (2)	0.071 (3)	0.122 (5)	0.004 (3)	−0.025 (3)	−0.019 (4)
C15	0.061 (3)	0.067 (3)	0.066 (3)	−0.026 (3)	−0.021 (3)	0.004 (3)

### Geometric parameters (Å, º)

**Table:**

Zr1—O1	1.921 (3)	C2—C3	1.361 (9)
Zr1—Cl1	2.4857 (12)	C2—H2	0.9400
Zr1—C3	2.496 (5)	C3—C4	1.392 (7)
Zr1—C9	2.504 (6)	C3—H3	0.9400
Zr1—C7	2.508 (5)	C4—C5	1.377 (6)
Zr1—C8	2.518 (5)	C4—H4	0.9400
Zr1—C4	2.526 (4)	C5—H5	0.9400
Zr1—C6	2.529 (5)	C6—C10	1.360 (9)
Zr1—C5	2.530 (4)	C6—C7	1.383 (9)
Zr1—C2	2.531 (5)	C6—H6	0.9400
Zr1—C10	2.534 (5)	C7—C8	1.374 (9)
Zr1—C1	2.541 (5)	C7—H7	0.9400
Zr2—O1	1.980 (3)	C8—C9	1.337 (9)
Zr2—O1^i^	1.980 (2)	C8—H8	0.9400
Zr2—C14	2.509 (5)	C9—C10	1.304 (9)
Zr2—C14^i^	2.509 (5)	C9—H9	0.9400
Zr2—C13	2.512 (5)	C10—H10	0.9400
Zr2—C13^i^	2.512 (5)	C11—C12	1.361 (8)
Zr2—C12^i^	2.523 (5)	C11—C15	1.362 (7)
Zr2—C12	2.523 (5)	C11—H11	0.9400
Zr2—C11^i^	2.532 (5)	C12—C13	1.340 (8)
Zr2—C11	2.532 (5)	C12—H12	0.9400
Zr2—C15	2.554 (5)	C13—C14	1.430 (7)
Zr2—C15^i^	2.554 (5)	C13—H13	0.9400
C1—C2	1.365 (9)	C14—C15	1.380 (7)
C1—C5	1.386 (7)	C14—H14	0.9400
C1—H1	0.9400	C15—H15	0.9400
			
O1—Zr1—Cl1	97.23 (8)	O1^i^—Zr2—C15	113.33 (16)
O1—Zr1—C3	96.9 (2)	C14—Zr2—C15	31.61 (17)
Cl1—Zr1—C3	133.18 (14)	C14^i^—Zr2—C15	148.31 (18)
O1—Zr1—C9	87.5 (2)	C13—Zr2—C15	52.66 (18)
Cl1—Zr1—C9	129.42 (19)	C13^i^—Zr2—C15	118.17 (19)
C3—Zr1—C9	95.6 (2)	C12^i^—Zr2—C15	111.8 (2)
O1—Zr1—C7	134.54 (16)	C12—Zr2—C15	51.47 (18)
Cl1—Zr1—C7	95.6 (2)	C11^i^—Zr2—C15	130.9 (2)
C3—Zr1—C7	105.3 (3)	C11—Zr2—C15	31.07 (16)
C9—Zr1—C7	51.8 (2)	O1—Zr2—C15^i^	113.33 (16)
O1—Zr1—C8	117.1 (2)	O1^i^—Zr2—C15^i^	78.61 (14)
Cl1—Zr1—C8	127.13 (19)	C14—Zr2—C15^i^	148.31 (18)
C3—Zr1—C8	83.6 (2)	C14^i^—Zr2—C15^i^	31.61 (17)
C9—Zr1—C8	30.9 (2)	C13—Zr2—C15^i^	118.17 (19)
C7—Zr1—C8	31.7 (2)	C13^i^—Zr2—C15^i^	52.66 (18)
O1—Zr1—C4	79.99 (13)	C12^i^—Zr2—C15^i^	51.47 (18)
Cl1—Zr1—C4	108.64 (13)	C12—Zr2—C15^i^	111.8 (2)
C3—Zr1—C4	32.18 (17)	C11^i^—Zr2—C15^i^	31.07 (16)
C9—Zr1—C4	121.7 (2)	C11—Zr2—C15^i^	130.9 (2)
C7—Zr1—C4	135.3 (2)	C15—Zr2—C15^i^	161.8 (2)
C8—Zr1—C4	115.67 (19)	Zr1—O1—Zr2	171.43 (15)
O1—Zr1—C6	109.1 (2)	C2—C1—C5	107.9 (6)
Cl1—Zr1—C6	80.37 (14)	C2—C1—Zr1	74.0 (3)
C3—Zr1—C6	134.8 (2)	C5—C1—Zr1	73.7 (3)
C9—Zr1—C6	51.1 (2)	C2—C1—H1	126.1
C7—Zr1—C6	31.9 (2)	C5—C1—H1	126.1
C8—Zr1—C6	51.88 (19)	Zr1—C1—H1	118.2
C4—Zr1—C6	166.7 (2)	C3—C2—C1	108.7 (5)
O1—Zr1—C5	98.69 (14)	C3—C2—Zr1	72.9 (3)
Cl1—Zr1—C5	81.19 (12)	C1—C2—Zr1	74.8 (3)
C3—Zr1—C5	52.61 (17)	C3—C2—H2	125.7
C9—Zr1—C5	148.0 (2)	C1—C2—H2	125.7
C7—Zr1—C5	126.34 (18)	Zr1—C2—H2	118.5
C8—Zr1—C5	126.85 (19)	C2—C3—C4	108.3 (6)
C4—Zr1—C5	31.62 (15)	C2—C3—Zr1	75.7 (3)
C6—Zr1—C5	148.3 (2)	C4—C3—Zr1	75.1 (3)
O1—Zr1—C2	128.0 (2)	C2—C3—H3	125.9
Cl1—Zr1—C2	115.3 (2)	C4—C3—H3	125.9
C3—Zr1—C2	31.4 (2)	Zr1—C3—H3	115.5
C9—Zr1—C2	99.7 (3)	C5—C4—C3	107.1 (5)
C7—Zr1—C2	83.5 (2)	C5—C4—Zr1	74.3 (2)
C8—Zr1—C2	74.7 (2)	C3—C4—Zr1	72.7 (3)
C4—Zr1—C2	52.37 (18)	C5—C4—H4	126.5
C6—Zr1—C2	115.3 (2)	C3—C4—H4	126.5
C5—Zr1—C2	52.14 (19)	Zr1—C4—H4	118.5
O1—Zr1—C10	83.03 (16)	C4—C5—C1	108.0 (5)
Cl1—Zr1—C10	100.4 (2)	C4—C5—Zr1	74.1 (2)
C3—Zr1—C10	125.5 (2)	C1—C5—Zr1	74.6 (3)
C9—Zr1—C10	30.0 (2)	C4—C5—H5	126.0
C7—Zr1—C10	51.76 (19)	C1—C5—H5	126.0
C8—Zr1—C10	50.7 (2)	Zr1—C5—H5	117.4
C4—Zr1—C10	147.9 (2)	C10—C6—C7	106.7 (6)
C6—Zr1—C10	31.2 (2)	C10—C6—Zr1	74.6 (3)
C5—Zr1—C10	177.53 (19)	C7—C6—Zr1	73.2 (3)
C2—Zr1—C10	125.4 (2)	C10—C6—H6	126.7
O1—Zr1—C1	129.66 (15)	C7—C6—H6	126.7
Cl1—Zr1—C1	85.3 (2)	Zr1—C6—H6	117.6
C3—Zr1—C1	52.2 (2)	C8—C7—C6	106.4 (6)
C9—Zr1—C1	128.6 (3)	C8—C7—Zr1	74.6 (3)
C7—Zr1—C1	94.7 (2)	C6—C7—Zr1	74.9 (3)
C8—Zr1—C1	99.5 (3)	C8—C7—H7	126.8
C4—Zr1—C1	52.37 (16)	C6—C7—H7	126.8
C6—Zr1—C1	120.8 (2)	Zr1—C7—H7	116.1
C5—Zr1—C1	31.73 (17)	C9—C8—C7	107.7 (6)
C2—Zr1—C1	31.2 (2)	C9—C8—Zr1	74.0 (3)
C10—Zr1—C1	146.2 (2)	C7—C8—Zr1	73.7 (3)
O1—Zr2—O1^i^	102.43 (15)	C9—C8—H8	126.2
O1—Zr2—C14	96.13 (17)	C7—C8—H8	126.2
O1^i^—Zr2—C14	84.03 (16)	Zr1—C8—H8	118.1
O1—Zr2—C14^i^	84.03 (16)	C10—C9—C8	109.9 (7)
O1^i^—Zr2—C14^i^	96.13 (17)	C10—C9—Zr1	76.3 (3)
C14—Zr2—C14^i^	179.7 (3)	C8—C9—Zr1	75.2 (4)
O1—Zr2—C13	128.40 (15)	C10—C9—H9	125.0
O1^i^—Zr2—C13	84.87 (16)	C8—C9—H9	125.0
C14—Zr2—C13	33.10 (17)	Zr1—C9—H9	115.5
C14^i^—Zr2—C13	146.71 (19)	C9—C10—C6	109.2 (6)
O1—Zr2—C13^i^	84.87 (16)	C9—C10—Zr1	73.7 (3)
O1^i^—Zr2—C13^i^	128.40 (15)	C6—C10—Zr1	74.2 (3)
C14—Zr2—C13^i^	146.71 (19)	C9—C10—H10	125.4
C14^i^—Zr2—C13^i^	33.10 (17)	C6—C10—H10	125.4
C13—Zr2—C13^i^	129.8 (3)	Zr1—C10—H10	118.5
O1—Zr2—C12^i^	113.78 (19)	C12—C11—C15	108.1 (6)
O1^i^—Zr2—C12^i^	126.25 (17)	C12—C11—Zr2	74.0 (3)
C14—Zr2—C12^i^	127.2 (2)	C15—C11—Zr2	75.3 (3)
C14^i^—Zr2—C12^i^	52.50 (18)	C12—C11—H11	125.9
C13—Zr2—C12^i^	100.6 (2)	C15—C11—H11	125.9
C13^i^—Zr2—C12^i^	30.87 (17)	Zr2—C11—H11	116.8
O1—Zr2—C12	126.24 (17)	C13—C12—C11	110.2 (5)
O1^i^—Zr2—C12	113.78 (19)	C13—C12—Zr2	74.1 (3)
C14—Zr2—C12	52.50 (18)	C11—C12—Zr2	74.8 (3)
C14^i^—Zr2—C12	127.2 (2)	C13—C12—H12	124.9
C13—Zr2—C12	30.87 (17)	C11—C12—H12	124.9
C13^i^—Zr2—C12	100.6 (2)	Zr2—C12—H12	118.0
C12^i^—Zr2—C12	74.9 (3)	C12—C13—C14	106.9 (5)
O1—Zr2—C11^i^	134.52 (16)	C12—C13—Zr2	75.0 (3)
O1^i^—Zr2—C11^i^	95.43 (17)	C14—C13—Zr2	73.4 (3)
C14—Zr2—C11^i^	127.4 (2)	C12—C13—H13	126.6
C14^i^—Zr2—C11^i^	52.42 (18)	C14—C13—H13	126.6
C13—Zr2—C11^i^	94.3 (2)	Zr2—C13—H13	117.2
C13^i^—Zr2—C11^i^	52.1 (2)	C15—C14—C13	106.2 (5)
C12^i^—Zr2—C11^i^	31.23 (18)	C15—C14—Zr2	76.0 (3)
C12—Zr2—C11^i^	81.2 (2)	C13—C14—Zr2	73.5 (3)
O1—Zr2—C11	95.43 (17)	C15—C14—H14	126.9
O1^i^—Zr2—C11	134.52 (16)	C13—C14—H14	126.9
C14—Zr2—C11	52.42 (18)	Zr2—C14—H14	116.0
C14^i^—Zr2—C11	127.4 (2)	C11—C15—C14	108.6 (5)
C13—Zr2—C11	52.1 (2)	C11—C15—Zr2	73.6 (3)
C13^i^—Zr2—C11	94.3 (2)	C14—C15—Zr2	72.4 (3)
C12^i^—Zr2—C11	81.2 (2)	C11—C15—H15	125.7
C12—Zr2—C11	31.23 (18)	C14—C15—H15	125.7
C11^i^—Zr2—C11	101.1 (3)	Zr2—C15—H15	120.1
O1—Zr2—C15	78.61 (14)		
			
Cl1—Zr1—O1—Zr2	21.7 (10)	Cl1—Zr1—C8—C9	−106.6 (5)
C3—Zr1—O1—Zr2	156.9 (10)	C3—Zr1—C8—C9	112.2 (5)
C9—Zr1—O1—Zr2	−107.7 (10)	C7—Zr1—C8—C9	−114.4 (6)
C7—Zr1—O1—Zr2	−83.6 (11)	C4—Zr1—C8—C9	109.4 (4)
C8—Zr1—O1—Zr2	−116.8 (10)	C6—Zr1—C8—C9	−76.0 (4)
C4—Zr1—O1—Zr2	129.4 (10)	C5—Zr1—C8—C9	144.3 (4)
C6—Zr1—O1—Zr2	−60.6 (10)	C2—Zr1—C8—C9	143.0 (6)
C5—Zr1—O1—Zr2	103.8 (10)	C10—Zr1—C8—C9	−35.7 (4)
C2—Zr1—O1—Zr2	151.9 (10)	C1—Zr1—C8—C9	162.1 (5)
C10—Zr1—O1—Zr2	−78.0 (10)	O1—Zr1—C8—C7	132.2 (5)
C1—Zr1—O1—Zr2	111.5 (10)	Cl1—Zr1—C8—C7	7.8 (6)
O1^i^—Zr2—O1—Zr1	−94.5 (10)	C3—Zr1—C8—C7	−133.4 (5)
C14—Zr2—O1—Zr1	−9.3 (10)	C9—Zr1—C8—C7	114.4 (6)
C14^i^—Zr2—O1—Zr1	170.5 (10)	C4—Zr1—C8—C7	−136.2 (4)
C13—Zr2—O1—Zr1	−1.2 (11)	C6—Zr1—C8—C7	38.4 (4)
C13^i^—Zr2—O1—Zr1	137.3 (10)	C5—Zr1—C8—C7	−101.3 (4)
C12^i^—Zr2—O1—Zr1	126.1 (10)	C2—Zr1—C8—C7	−102.6 (5)
C12—Zr2—O1—Zr1	37.8 (11)	C10—Zr1—C8—C7	78.7 (4)
C11^i^—Zr2—O1—Zr1	154.8 (10)	C1—Zr1—C8—C7	−83.5 (5)
C11—Zr2—O1—Zr1	43.4 (10)	C7—C8—C9—C10	2.5 (7)
C15—Zr2—O1—Zr1	17.2 (10)	Zr1—C8—C9—C10	69.1 (4)
C15^i^—Zr2—O1—Zr1	−177.3 (10)	C7—C8—C9—Zr1	−66.6 (4)
O1—Zr1—C1—C2	100.0 (5)	O1—Zr1—C9—C10	80.4 (5)
Cl1—Zr1—C1—C2	−164.5 (4)	Cl1—Zr1—C9—C10	−16.8 (6)
C3—Zr1—C1—C2	36.5 (4)	C3—Zr1—C9—C10	177.1 (5)
C9—Zr1—C1—C2	−26.0 (5)	C7—Zr1—C9—C10	−77.8 (5)
C7—Zr1—C1—C2	−69.2 (5)	C8—Zr1—C9—C10	−115.3 (7)
C8—Zr1—C1—C2	−37.6 (5)	C4—Zr1—C9—C10	157.0 (4)
C4—Zr1—C1—C2	77.6 (4)	C6—Zr1—C9—C10	−36.7 (4)
C6—Zr1—C1—C2	−88.7 (4)	C5—Zr1—C9—C10	−177.2 (4)
C5—Zr1—C1—C2	114.6 (6)	C2—Zr1—C9—C10	−151.5 (5)
C10—Zr1—C1—C2	−62.9 (6)	C1—Zr1—C9—C10	−138.1 (5)
O1—Zr1—C1—C5	−14.6 (5)	O1—Zr1—C9—C8	−164.2 (5)
Cl1—Zr1—C1—C5	80.9 (4)	Cl1—Zr1—C9—C8	98.5 (5)
C3—Zr1—C1—C5	−78.1 (4)	C3—Zr1—C9—C8	−67.6 (5)
C9—Zr1—C1—C5	−140.6 (4)	C7—Zr1—C9—C8	37.6 (4)
C7—Zr1—C1—C5	176.1 (4)	C4—Zr1—C9—C8	−87.6 (5)
C8—Zr1—C1—C5	−152.2 (4)	C6—Zr1—C9—C8	78.6 (5)
C4—Zr1—C1—C5	−37.1 (3)	C5—Zr1—C9—C8	−61.8 (6)
C6—Zr1—C1—C5	156.7 (3)	C2—Zr1—C9—C8	−36.1 (5)
C2—Zr1—C1—C5	−114.6 (6)	C10—Zr1—C9—C8	115.3 (7)
C10—Zr1—C1—C5	−177.5 (5)	C1—Zr1—C9—C8	−22.8 (6)
C5—C1—C2—C3	0.9 (6)	C8—C9—C10—C6	−2.0 (7)
Zr1—C1—C2—C3	−65.5 (4)	Zr1—C9—C10—C6	66.3 (4)
C5—C1—C2—Zr1	66.5 (4)	C8—C9—C10—Zr1	−68.3 (4)
O1—Zr1—C2—C3	9.6 (6)	C7—C6—C10—C9	0.7 (6)
Cl1—Zr1—C2—C3	132.7 (4)	Zr1—C6—C10—C9	−66.0 (4)
C9—Zr1—C2—C3	−84.8 (4)	C7—C6—C10—Zr1	66.7 (4)
C7—Zr1—C2—C3	−134.2 (5)	O1—Zr1—C10—C9	−97.0 (5)
C8—Zr1—C2—C3	−103.1 (5)	Cl1—Zr1—C10—C9	166.9 (5)
C4—Zr1—C2—C3	38.0 (3)	C3—Zr1—C10—C9	−3.5 (6)
C6—Zr1—C2—C3	−136.2 (4)	C7—Zr1—C10—C9	77.9 (5)
C5—Zr1—C2—C3	78.3 (4)	C8—Zr1—C10—C9	36.8 (4)
C10—Zr1—C2—C3	−101.8 (4)	C4—Zr1—C10—C9	−38.6 (6)
C1—Zr1—C2—C3	115.5 (6)	C6—Zr1—C10—C9	116.0 (6)
O1—Zr1—C2—C1	−105.9 (5)	C5—Zr1—C10—C9	37 (6)
Cl1—Zr1—C2—C1	17.1 (5)	C2—Zr1—C10—C9	35.3 (7)
C3—Zr1—C2—C1	−115.5 (6)	C1—Zr1—C10—C9	69.8 (8)
C9—Zr1—C2—C1	159.7 (4)	O1—Zr1—C10—C6	147.0 (5)
C7—Zr1—C2—C1	110.3 (5)	Cl1—Zr1—C10—C6	50.8 (4)
C8—Zr1—C2—C1	141.4 (5)	C3—Zr1—C10—C6	−119.5 (5)
C4—Zr1—C2—C1	−77.6 (4)	C9—Zr1—C10—C6	−116.0 (6)
C6—Zr1—C2—C1	108.2 (4)	C7—Zr1—C10—C6	−38.2 (4)
C5—Zr1—C2—C1	−37.3 (3)	C8—Zr1—C10—C6	−79.2 (4)
C10—Zr1—C2—C1	142.6 (4)	C4—Zr1—C10—C6	−154.6 (4)
C1—C2—C3—C4	−1.9 (6)	C5—Zr1—C10—C6	−79 (6)
Zr1—C2—C3—C4	−68.7 (3)	C2—Zr1—C10—C6	−80.7 (6)
C1—C2—C3—Zr1	66.8 (4)	C1—Zr1—C10—C6	−46.2 (8)
O1—Zr1—C3—C2	−172.4 (4)	O1—Zr2—C11—C12	−171.3 (4)
Cl1—Zr1—C3—C2	−65.7 (5)	O1^i^—Zr2—C11—C12	−57.9 (5)
C9—Zr1—C3—C2	99.5 (5)	C14—Zr2—C11—C12	−77.7 (4)
C7—Zr1—C3—C2	47.7 (5)	C14^i^—Zr2—C11—C12	102.1 (4)
C8—Zr1—C3—C2	71.0 (5)	C13—Zr2—C11—C12	−35.5 (4)
C4—Zr1—C3—C2	−113.7 (5)	C13^i^—Zr2—C11—C12	103.4 (4)
C6—Zr1—C3—C2	61.9 (6)	C12^i^—Zr2—C11—C12	75.4 (5)
C5—Zr1—C3—C2	−76.7 (4)	C11^i^—Zr2—C11—C12	51.3 (4)
C10—Zr1—C3—C2	101.3 (5)	C15—Zr2—C11—C12	−114.1 (6)
C1—Zr1—C3—C2	−36.3 (4)	C15^i^—Zr2—C11—C12	61.2 (5)
O1—Zr1—C3—C4	−58.6 (4)	O1—Zr2—C11—C15	−57.2 (4)
Cl1—Zr1—C3—C4	48.1 (5)	O1^i^—Zr2—C11—C15	56.2 (5)
C9—Zr1—C3—C4	−146.7 (4)	C14—Zr2—C11—C15	36.4 (3)
C7—Zr1—C3—C4	161.4 (4)	C14^i^—Zr2—C11—C15	−143.8 (3)
C8—Zr1—C3—C4	−175.2 (4)	C13—Zr2—C11—C15	78.6 (4)
C6—Zr1—C3—C4	175.6 (4)	C13^i^—Zr2—C11—C15	−142.4 (4)
C5—Zr1—C3—C4	37.1 (3)	C12^i^—Zr2—C11—C15	−170.4 (5)
C2—Zr1—C3—C4	113.7 (5)	C12—Zr2—C11—C15	114.1 (6)
C10—Zr1—C3—C4	−145.0 (3)	C11^i^—Zr2—C11—C15	165.4 (5)
C1—Zr1—C3—C4	77.4 (4)	C15^i^—Zr2—C11—C15	175.32 (16)
C2—C3—C4—C5	2.1 (6)	C15—C11—C12—C13	−2.0 (6)
Zr1—C3—C4—C5	−67.0 (3)	Zr2—C11—C12—C13	66.3 (4)
C2—C3—C4—Zr1	69.1 (4)	C15—C11—C12—Zr2	−68.2 (4)
O1—Zr1—C4—C5	−125.4 (3)	O1—Zr2—C12—C13	−105.9 (4)
Cl1—Zr1—C4—C5	−31.1 (3)	O1^i^—Zr2—C12—C13	22.0 (4)
C3—Zr1—C4—C5	114.0 (5)	C14—Zr2—C12—C13	−39.3 (4)
C9—Zr1—C4—C5	153.9 (3)	C14^i^—Zr2—C12—C13	140.8 (3)
C7—Zr1—C4—C5	88.1 (4)	C13^i^—Zr2—C12—C13	162.6 (3)
C8—Zr1—C4—C5	119.2 (4)	C12^i^—Zr2—C12—C13	145.4 (5)
C6—Zr1—C4—C5	100.3 (10)	C11^i^—Zr2—C12—C13	114.1 (4)
C2—Zr1—C4—C5	77.0 (4)	C11—Zr2—C12—C13	−116.7 (6)
C10—Zr1—C4—C5	175.4 (3)	C15—Zr2—C12—C13	−79.7 (4)
C1—Zr1—C4—C5	37.2 (3)	C15^i^—Zr2—C12—C13	108.8 (4)
O1—Zr1—C4—C3	120.6 (4)	O1—Zr2—C12—C11	10.8 (5)
Cl1—Zr1—C4—C3	−145.1 (4)	O1^i^—Zr2—C12—C11	138.7 (4)
C9—Zr1—C4—C3	39.9 (5)	C14—Zr2—C12—C11	77.4 (4)
C7—Zr1—C4—C3	−25.9 (5)	C14^i^—Zr2—C12—C11	−102.5 (4)
C8—Zr1—C4—C3	5.2 (5)	C13—Zr2—C12—C11	116.7 (6)
C6—Zr1—C4—C3	−13.7 (11)	C13^i^—Zr2—C12—C11	−80.7 (4)
C5—Zr1—C4—C3	−114.0 (5)	C12^i^—Zr2—C12—C11	−97.9 (5)
C2—Zr1—C4—C3	−37.0 (4)	C11^i^—Zr2—C12—C11	−129.2 (4)
C10—Zr1—C4—C3	61.4 (5)	C15—Zr2—C12—C11	37.0 (4)
C1—Zr1—C4—C3	−76.8 (4)	C15^i^—Zr2—C12—C11	−134.5 (4)
C3—C4—C5—C1	−1.6 (5)	C11—C12—C13—C14	0.3 (6)
Zr1—C4—C5—C1	−67.4 (3)	Zr2—C12—C13—C14	67.0 (3)
C3—C4—C5—Zr1	65.9 (3)	C11—C12—C13—Zr2	−66.7 (4)
C2—C1—C5—C4	0.4 (6)	O1—Zr2—C13—C12	98.3 (4)
Zr1—C1—C5—C4	67.1 (3)	O1^i^—Zr2—C13—C12	−159.9 (4)
C2—C1—C5—Zr1	−66.7 (4)	C14—Zr2—C13—C12	113.2 (5)
O1—Zr1—C5—C4	54.3 (3)	C14^i^—Zr2—C13—C12	−66.5 (5)
Cl1—Zr1—C5—C4	150.3 (3)	C13^i^—Zr2—C13—C12	−22.4 (3)
C3—Zr1—C5—C4	−37.8 (3)	C12^i^—Zr2—C13—C12	−33.9 (5)
C9—Zr1—C5—C4	−44.9 (5)	C11^i^—Zr2—C13—C12	−64.8 (4)
C7—Zr1—C5—C4	−119.2 (4)	C11—Zr2—C13—C12	36.0 (3)
C8—Zr1—C5—C4	−79.4 (4)	C15—Zr2—C13—C12	75.5 (4)
C6—Zr1—C5—C4	−154.6 (4)	C15^i^—Zr2—C13—C12	−85.7 (4)
C2—Zr1—C5—C4	−77.7 (4)	O1—Zr2—C13—C14	−14.9 (4)
C10—Zr1—C5—C4	−80 (6)	O1^i^—Zr2—C13—C14	86.9 (3)
C1—Zr1—C5—C4	−114.4 (5)	C14^i^—Zr2—C13—C14	−179.7 (3)
O1—Zr1—C5—C1	168.7 (4)	C13^i^—Zr2—C13—C14	−135.6 (4)
Cl1—Zr1—C5—C1	−95.3 (4)	C12^i^—Zr2—C13—C14	−147.1 (4)
C3—Zr1—C5—C1	76.6 (4)	C12—Zr2—C13—C14	−113.2 (5)
C9—Zr1—C5—C1	69.4 (6)	C11^i^—Zr2—C13—C14	−178.0 (4)
C7—Zr1—C5—C1	−4.8 (5)	C11—Zr2—C13—C14	−77.2 (4)
C8—Zr1—C5—C1	35.0 (5)	C15—Zr2—C13—C14	−37.7 (3)
C4—Zr1—C5—C1	114.4 (5)	C15^i^—Zr2—C13—C14	161.1 (3)
C6—Zr1—C5—C1	−40.2 (6)	C12—C13—C14—C15	1.5 (6)
C2—Zr1—C5—C1	36.7 (4)	Zr2—C13—C14—C15	69.6 (3)
C10—Zr1—C5—C1	34 (6)	C12—C13—C14—Zr2	−68.1 (4)
O1—Zr1—C6—C10	−34.9 (5)	O1—Zr2—C14—C15	56.4 (3)
Cl1—Zr1—C6—C10	−129.3 (5)	O1^i^—Zr2—C14—C15	158.3 (3)
C3—Zr1—C6—C10	86.6 (6)	C14^i^—Zr2—C14—C15	−72.5 (5)
C9—Zr1—C6—C10	35.2 (4)	C13—Zr2—C14—C15	−111.9 (5)
C7—Zr1—C6—C10	113.2 (6)	C13^i^—Zr2—C14—C15	−33.6 (6)
C8—Zr1—C6—C10	75.0 (4)	C12^i^—Zr2—C14—C15	−69.8 (5)
C4—Zr1—C6—C10	96.8 (12)	C12—Zr2—C14—C15	−75.4 (3)
C5—Zr1—C6—C10	175.4 (4)	C11^i^—Zr2—C14—C15	−109.4 (4)
C2—Zr1—C6—C10	117.1 (5)	C11—Zr2—C14—C15	−35.7 (3)
C1—Zr1—C6—C10	152.1 (5)	C15^i^—Zr2—C14—C15	−144.9 (5)
O1—Zr1—C6—C7	−148.1 (5)	O1—Zr2—C14—C13	168.3 (3)
Cl1—Zr1—C6—C7	117.5 (5)	O1^i^—Zr2—C14—C13	−89.8 (4)
C3—Zr1—C6—C7	−26.6 (7)	C14^i^—Zr2—C14—C13	39.4 (4)
C9—Zr1—C6—C7	−78.0 (4)	C13^i^—Zr2—C14—C13	78.4 (7)
C8—Zr1—C6—C7	−38.2 (4)	C12^i^—Zr2—C14—C13	42.1 (5)
C4—Zr1—C6—C7	−16.4 (13)	C12—Zr2—C14—C13	36.5 (3)
C5—Zr1—C6—C7	62.2 (6)	C11^i^—Zr2—C14—C13	2.5 (5)
C2—Zr1—C6—C7	3.9 (6)	C11—Zr2—C14—C13	76.2 (4)
C10—Zr1—C6—C7	−113.2 (6)	C15—Zr2—C14—C13	111.9 (5)
C1—Zr1—C6—C7	38.9 (5)	C15^i^—Zr2—C14—C13	−32.9 (5)
C10—C6—C7—C8	0.8 (6)	C12—C11—C15—C14	2.9 (6)
Zr1—C6—C7—C8	68.5 (4)	Zr2—C11—C15—C14	−64.4 (3)
C10—C6—C7—Zr1	−67.7 (4)	C12—C11—C15—Zr2	67.4 (4)
O1—Zr1—C7—C8	−67.8 (6)	C13—C14—C15—C11	−2.7 (5)
Cl1—Zr1—C7—C8	−173.8 (5)	Zr2—C14—C15—C11	65.2 (4)
C3—Zr1—C7—C8	48.5 (5)	C13—C14—C15—Zr2	−67.9 (3)
C9—Zr1—C7—C8	−36.5 (4)	O1—Zr2—C15—C11	121.4 (4)
C4—Zr1—C7—C8	62.5 (5)	O1^i^—Zr2—C15—C11	−139.8 (4)
C6—Zr1—C7—C8	−112.2 (6)	C14—Zr2—C15—C11	−116.2 (5)
C5—Zr1—C7—C8	103.1 (4)	C14^i^—Zr2—C15—C11	63.3 (5)
C2—Zr1—C7—C8	71.3 (5)	C13—Zr2—C15—C11	−76.6 (4)
C10—Zr1—C7—C8	−75.0 (4)	C13^i^—Zr2—C15—C11	43.6 (5)
C1—Zr1—C7—C8	100.5 (5)	C12^i^—Zr2—C15—C11	10.2 (5)
O1—Zr1—C7—C6	44.4 (6)	C12—Zr2—C15—C11	−37.2 (4)
Cl1—Zr1—C7—C6	−61.5 (5)	C11^i^—Zr2—C15—C11	−19.1 (6)
C3—Zr1—C7—C6	160.8 (5)	C15^i^—Zr2—C15—C11	−11.4 (4)
C9—Zr1—C7—C6	75.7 (4)	O1—Zr2—C15—C14	−122.3 (3)
C8—Zr1—C7—C6	112.2 (6)	O1^i^—Zr2—C15—C14	−23.6 (4)
C4—Zr1—C7—C6	174.7 (4)	C14^i^—Zr2—C15—C14	179.5 (5)
C5—Zr1—C7—C6	−144.7 (4)	C13—Zr2—C15—C14	39.6 (3)
C2—Zr1—C7—C6	−176.4 (5)	C13^i^—Zr2—C15—C14	159.9 (4)
C10—Zr1—C7—C6	37.3 (4)	C12^i^—Zr2—C15—C14	126.4 (4)
C1—Zr1—C7—C6	−147.2 (5)	C12—Zr2—C15—C14	79.0 (4)
C6—C7—C8—C9	−2.0 (6)	C11^i^—Zr2—C15—C14	97.1 (4)
Zr1—C7—C8—C9	66.7 (4)	C11—Zr2—C15—C14	116.2 (5)
C6—C7—C8—Zr1	−68.7 (4)	C15^i^—Zr2—C15—C14	104.9 (3)
O1—Zr1—C8—C9	17.8 (5)		

Symmetry code: (i) −*x*+1, *y*, −*z*+1/2.
